# Impact of the Economic Status of the Patient’s Country of Residence on the Outcome of Oncology Clinical Trials

**DOI:** 10.1093/oncolo/oyab060

**Published:** 2022-02-26

**Authors:** Saki Nishiyama, Mamoru Narukawa

**Affiliations:** Graduate School of Pharmaceutical Sciences, Kitasato University, Tokyo, Japan; Graduate School of Pharmaceutical Sciences, Kitasato University, Tokyo, Japan

**Keywords:** clinical trial, drug development, economic status, medical oncology, meta-analysis

## Abstract

**Background:**

Prolongation of overall survival (OS) is commonly evaluated as a primary endpoint in confirmative oncology clinical trials; however, it is potentially affected by subsequent treatments carried out in practice. To design and implement multi-regional clinical trials properly, we compared survival outcomes between Organisation for Economic Co-operation and Development (OECD) and non-OECD countries.

**Materials and Methods:**

Individual patient data from industry-sponsored multi-regional phase III oncology trials were obtained from the Project Data Sphere. Patients of each arm were divided into several subgroups based on race and country where patients were enrolled. We defined the member countries of the OECD. Cox regression analysis was conducted to estimate the hazard ratio (HR) for progression-free survival (PFS) and OS between the different subgroups in each trial, followed by a meta-analysis to estimate the summary HR and its confidence interval with a random-effect model.

**Results:**

Eleven arms from 10 clinical trials were eligible for the analysis. No statistically significant difference was observed in PFS and OS between Caucasian and Asian. A prolongation of OS was observed in patients enrolled in the OECD group compared with non-OECD group, while no statistically significant difference was observed in PFS.

**Conclusion:**

The economic status and healthcare environment of countries where patients reside have an impact on the outcome of OS. Clinical trial sponsors are recommended to consider carefully how to properly design oncology clinical trials including the selection of countries and data management of subsequent treatments.

## Teaser

Because more multi-regional clinical trials will be conducted in pursuit of new oncology drugs, it is important to understand how socio-economic factors and health care environment affect clinical endpoints in different regions and countries. This article examines how ethnic factors, especially the economic status of countries, an important extrinsic factor, affect survival outcome in clinical trials of oncology drugs.

**Implications for Practice**

The purpose of this study was to evaluate the impact of economic status of countries where patients were enrolled on the outcome of oncology clinical trials. The results indicate that overall survival was prolonged in countries where patients had favorable economic status while progression-free survival did not show a statistically significant difference. In oncology clinical trials, economic status is considered to have an impact on the treatment during post-progression survival. Clinical trial sponsors should consider the economic status of countries when planning new clinical trials, and design the way to collect treatment information during the post-progression survival period.

## Introduction

In oncology drug development, clinical benefit such as prolongation of survival is commonly evaluated as the primary endpoint in confirmative clinical trials. Overall survival (OS), which is considered the most reliable endpoint in oncology drug trials, is defined as the time from randomization until death from any cause. Subsequent treatments during the period of post-progression survival (PPS) are often carried out in practice; in fact, Imai et al^1^ reported that PPS was strongly associated with OS after early-line treatment. OS has several advantages including being precise, easy to measure and with no bias, but at the same time, it needs longer follow-up periods and may be affected by subsequent cancer therapies after completion of the test/control treatment.

A recent example is the JAVELIN Lung 200 study, a multicenter, open-label, randomized, phase III trial conducted in 31 countries to compare avelumab with docetaxel in patients with platinum-treated advanced non–small cell lung cancer, where it was reported that the benefit of OS was not demonstrated.^2^ The median OS of docetaxel in this trial was longer than that seen in other trials of immune checkpoint inhibitors (ICIs).^3-5^ Post hoc analyses of this trial revealed that patients who received a subsequent ICI treatment tended to survive longer than those who did not, in both experimental and comparator treatment arms,^6^ and that the proportion of patients receiving subsequent ICIs increased year after year especially in the control arm, reflecting the increased availability of ICIs in different countries. Another example is the AVAGAST study,^7^ which was a randomized, double-blind, phase III trial to evaluate the efficacy of adding bevacizumab to capecitabine plus cisplatin in the first-line treatment of patients with advanced gastric cancer. This study revealed that patients enrolled in the Asian region showed longer OS than those enrolled in Europe, South America, and the US. The prolongation of OS in patients enrolled in control arms, especially in Asia, was also reported in several other trials.^8-10^ These reports pointed out the high proportion of post-progression treatment in specific regions.

In recent years, many new oncology drugs are actively being developed worldwide. The proportion of multi-regional clinical trials (MRCTs), which are one of the most effective approaches in the global development of new pharmaceutical products, has been increasing over the past decade.^11^ Over 80% of oncology phase III trials that were initiated by the top 10 pharmaceutical companies between 2008 and 2017 were MRCTs.^11^ The International Council for Harmonisation of Technical Requirements for Pharmaceuticals for Human Use (ICH) E5 guideline^12^ in 1998 defined “ethnic factors” as those factors relating to genetic and physiologic (intrinsic) and cultural and environmental (extrinsic) characteristics of a population, that may impact the efficacy and/or safety of a drug. Medical practice and socio-economic status are examples of extrinsic ethnic factors. The ICH E17 guideline^13^ published in 2017 also emphasizes the importance of considering the differences among regions during the planning phase of MRCTs.

In regards to socio-economic status, Vrdoljak et al^14^ reported that cancer mortality is correlated with the expenditures on oncology drugs in real-world settings. In Europe, there was a large difference in expenditure on oncology drugs between Western Europe and Central and Eastern Europe, which led to disparity in the mortality-to-incidence (M/I) ratio. While the M/I ratio was affected by several factors, such as prevention, diagnosis, surgery, and radiation, one of the most important reasons that caused the disparity was better access to new, innovative, often expensive but effective oncology drugs in Western Europe.

The objective of the present analysis was to examine how the economic status of countries had an impact on the survival outcome of clinical trials of oncology drugs. As it is expected that more MRCTs will be conducted in pursuit of new oncology drugs, it is important to understand how socioeconomic factors and the healthcare environment affect the clinical endpoints in different regions/countries to make MRCTs more efficient and effective.

## Materials and Methods

### Data Sources

We obtained patient-level raw data of clinical trials from Project Data Sphere (PDS),^15^ a non-profit organization that allows registered researchers to access and analyze de-identified patient-level data from clinical trials in oncology. Eligible trials meeting the following criteria were identified and selected: (1) trials with an independent dataset in the database; (2) industry-sponsored trials; (3) multi-regional trials; (4) phase III trials; and (5) trials with information on race, country where a patient was enrolled, progression-free survival (PFS) and OS was available.

### Data Collection

The following information was identified for the selected trials: PDS UID, ClinicalTrials.gov identifier, sponsor, tumor type, arm (experimental/comparator), number of patients, and treatment drug(s). For each of the patients in the trials, the following data were collected from the dataset (where available): race, age at diagnosis, gender, an Eastern Cooperative Oncology Group (ECOG) or the World Health Organisation (WHO) performance status at diagnosis, the country where the patient was enrolled, PFS, and OS. The start year of the trial, primary endpoint(s), stage of cancer, and treatment line were investigated based on the information on the year first posted, outcome measures, and criteria in the ClinicalTrials.gov, respectively. Because the categorization of the race was different among sponsors, we defined Caucasian and White as “Caucasian,” Black, Black African, and African American as “Black,” Asian, east Asian and west Asian as “Asian” and all other categories and missing information as “Others.” PFS was defined as the time from randomization until objective tumor progression or death, whichever occurred first. OS was defined as the time from randomization until death from any cause. If PFS and/or OS data were not included in the dataset, we calculated them using the date of randomization, disease progression, and death.

### Definition of Analytical Groups

To evaluate the impact of the healthcare environment on the survival outcomes of oncology drugs, subjects in each arm (comparator arm and experimental arm) were divided into several subgroups based on race and countries where they were enrolled. For race, we classified the subjects into 4 groups: Caucasian, Asian, Black, and Others. Because the number of subjects of Black and Others was limited, we used Caucasian and Asian for the analysis. In regards to the country, we referenced to the Organisation for Economic Co-operation and Development (OECD),^16^ an international organization working to build better policies for better lives. We divided the patients into 2 subgroups based on the country where they were enrolled: the OECD group and the non-OECD group. We referred to the OECD membership status as of 2006.

### Statistical Analysis

Cox regression analysis was conducted to estimate the hazard ratio (HR) and its confidence interval (95% CI) for PFS and OS between Caucasian and Asian, and between OECD and non-OECD groups. We conducted a meta-analysis to estimate the summary HR with a random effect model. Heterogeneity between trials was evaluated using the I^2^ statistic. All the analysis was conducted using the R package metafor (R Foundation for Statistical Computing, Vienna, Austria).

## Results

**Figure 1.**
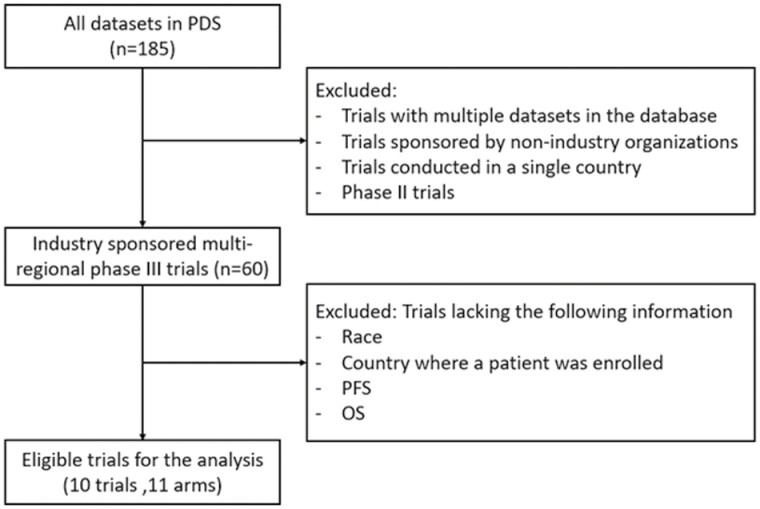
Flow diagram for trial selection in the Project Data Sphere platform. Abbreviations: OS, overall survival; PFS, progression-free survival.

Ten clinical trials met the eligible criteria, as shown in Fig. 1. For one of the trials, the data from both the comparator and the experimental arms were available, but for the other 9 trials, the data of the comparator arm were only available. Thus, we used data of these 11 arms for the analysis. Table 1 shows the list of trials used in the present study. The countries where patients were enrolled, their OECD membership status and the number of patients enrolled from each country are shown in Table 2. The characteristics of subgroups are shown in Table 3. Among 4437 patients in total, 2873 patients were enrolled in the OECD group.

**Table 1.** Summary of the eligible trials.

PDS UID	ClinicalTrials.gov Identifier	Sponsor	Start year of trial	Tumor type	Stage	Arm	Primary endpoint(s)	No. of patients	Treatment line	Treatment drugs
Colorec_AstraZe_2006_78	NCT00384176	AstraZeneca	2006	Colorectal	Stage IV (metastasis)	Comparator	PFS	690	1^st^	Bevacizumab + FOLFOX
Prostat_AstraZe_2008_103	NCT00626548	AstraZeneca	2008	Prostate	No metastases	Comparator	OS/PFS	716	1^st^	Placebo
Prostat_AstraZe_2008_104	NCT00617669	AstraZeneca	2008	Prostate	Metastasis (bone)	Comparator	OS	528	1^st^	Placebo + Docetaxel
Prostat_AstraZe_2009_144	NCT00554229	AstraZeneca	2007	Prostate	Metastasis (bone)	Comparator	OS	295	1^st^	Placebo
LungNo_ELiLill_2006_116	NCT00363415	Eli Lilly	2006	SCLC	Extensive stage	Comparator	OS	455	1^st^	Etoposide + Carboplatin
LungNo_EliLill_2008_148	NCT00686959	Eli Lilly	2008	NSCLC	Stage IIIA or IIIB	Comparator	OS	297	1^st^	Etoposide + Cisplatin and concurrent thoracic radiation therapy
HeadNe_EliLill_2006_150	NCT00415194	Eli Lilly	2006	Head and Neck	Recurrent or Metastasis	Comparator	OS	397	1^st^	Placebo + Cisplatin
Breast_EliLill_2008_168	NCT00703326	Eli Lilly	2008	Breast	Metastasis or locally recurrent	Comparator	PFS	385	1^st^	Placebo + Docetaxel
LungNo_EliLill_2010_272	NCT00981058	Eli Lilly	2009	NSCLC	Stage IV	Comparator	OS	548	1^st^	Gemcitabine + Cisplatin
Pancrea_ClovisO_2010_186	NCT01124786	Clovis Oncology	2010	Pancreatic	Metastasis	Comparator	OS	185	1^st^	Gemcitabine
Pancrea_ClovisO_2010_186	NCT01124786	Clovis Oncology	2010	Pancreatic	Metastasis	Experimental	OS	182	1^st^	CO-1.01 (Gemcitabine Elaidate)

NSCLC, non–small cell lung cancer; OS, overall survival; PFS, progression-free survival; SCLC, small cell lung cancer.

**Table 2.** OECD membership status of countries and number of enrolled patients.

Country	OECD membership status(as of 2006)	Number of patients (total of 11 arms)
Argentina	N	28
Australia	Y	234
Austria	Y	43
Belgium	Y	163
Brazil	N	161
Canada	Y	189
Chile	N	5
China	N	151
Colombia	N	2
Croatia	N	4
Czech Republic	Y	110
Denmark	Y	27
Egypt	N	15
Finland	Y	30
France	Y	212
Germany	Y	224
Greece	Y	25
Hungary	Y	154
India	N	189
Ireland	Y	9
Israel	N	21
Italy	Y	111
Japan	Y	95
Korea, Republic of	Y	113
Latvia	N	12
Lebanon	N	14
Malta	N	8
Mexico	Y	10
Netherlands	Y	72
New zealand	Y	8
Norway	Y	7
Peru	N	39
Philippines	N	17
Poland	Y	143
Portugal	Y	30
Romania	N	105
Russia	N	355
Serbia	N	34
Singapore	N	3
Slovakia	Y	23
South Africa	N	77
Spain	Y	239
Sweden	Y	37
Switzerland	Y	4
Taiwan	N	67
Thailand	N	16
Turkey	Y	26
Ukraine	N	188
United Kingdom	Y	156
US	Y	420
Vietnam	N	12

OECD, the Organisation for Economic Co-operation and Development.

**Table 3.** Baseline characteristics of study participants.

				Race				OECD/non-OECD			
		Overall		Caucasian		Asian		OECD group		Non-OECD group	
Average number of subjects in each study, total [min-max]											
		403	[182-690]	314	[157-644]	62.9	[2-142]	261	[88-484]	142	[67-213]
Race											
	Caucasian	314	[157-644]		-		-	228	[63-474]	86.5	[8-190]
	Black	8.73	[0-22]		-		-	4.82	[0-12]	3.91	[0-14]
	Asian	62.9	[2-142]		-		-	18.7	[2-72]	44.2	[0-133]
	Other	17.1	[5-44]		-		-	9.64	[1-43]	7.64	[0-40]
Median proportion in each study [25%-75% percentile]											
Age, years											
	18-64	60.4	[45.3-68.2]	64.4	[46.8-68.8]	65.6	[25.7-79.0]	56.4	[37.8-68.2]	72.8	[39.0-78.8]
	65-74	29.6	[25.1-38.6]	29.5	[24.2-35.9]	27.0	[18.4-45.4]	32.4	[28.6-41.7]	23.8	[18.4-41.4]
	75 -	8.01	[4.41-15.0]	7.64	[3.46-15.1]	1.35	[0-16.8]	10.2	[3.85-20.9]	4.26	[1.27-16.9]
Sex^a^											
	Male	60.0	[59.6-78.1]	59.9	[48.4-71.5]	64.9	[23.2-75.4]	67.0	[60.8-75.4]	63.9	[56.1-85.4]
	Female	40.0	[21.9-40.4]	40.1	[28.5-51.6]	35.1	[24.6-76.8]	33.0	[24.6-39.2]	36.1	[14.6-43.9]
ECOG/WHO Performance Status^b^											
	Unknown	0	[0-0.18]	0	[0-0]	0	[0-0]	0	[0-0.24]	0	[0-0]
	0	52.6	[32.8-48.8]	53.4	[33.9-62.5]	57.5	[19.0-74.7]	53.3	[39.3-64.5]	38.3	[24.9-64.2]
	1	47.4	[37.1-75.7]	45.1	[37.0-57.1]	10.6	[10.1-11.0]	45.3	[35.1-56.0]	61.7	[35.3-65.3]
	2	0	[0-0.55]	0	[0-0.16]	0	[0-0]	0	[0-0.47]	0	[0-0]
	3	0	[0-0]	0	[0-0]	0	[0-0]	0	[0-0]	0	[0-0]

a Prostat_AstraZe_2008_103, Prostat_AstraZe_2008_104, Prostat_AstraZe_2009_144, and Breast_EliLill_2008_168 were excluded because these trials included only male or female.

b LungNo_EliLill_2006_116 and HeadNe_EliLill_2006_150 were excluded because these trials collected performance status 0 and 1 as a same category.

Abbreviations: ECOG, an Eastern Cooperative Oncology Group; OECD, the Organisation for Economic Co-operation and Development; WHO, the World Health Organisation.

### Comparison of PFS and OS Between Caucasian and Asian

**Figure 2.**
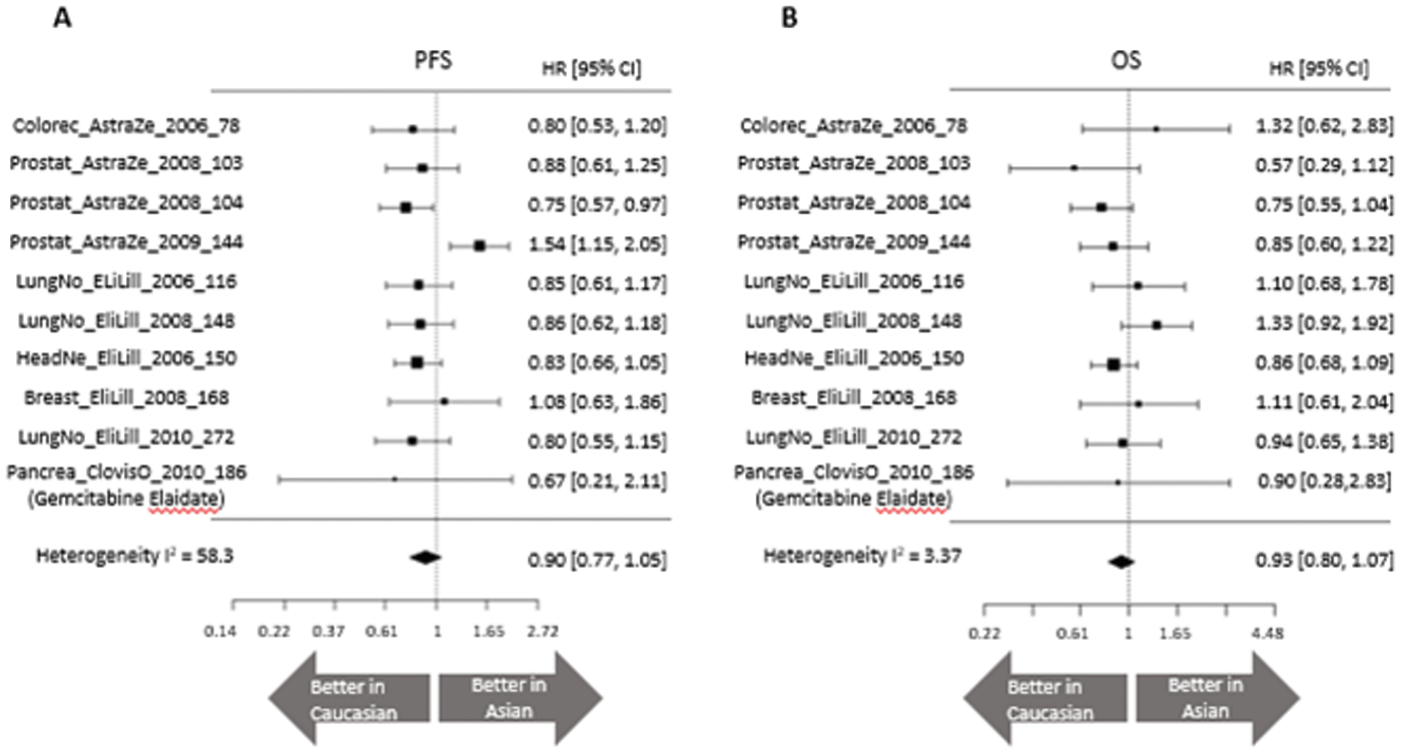
Forest plot of HRs comparing Caucasian and Asian for (**A**) PFS and (**B**) OS. Pancrea_ClovisO_2010_186 (Gemcitabine) was excluded because there was no Asian. CI, confidence interval; HR, hazard ratio; OS, overall survival; PFS, progression-free survival.

We calculated the HR for PFS and OS between Caucasian and Asian for each of the clinical trial data based on Cox regression analysis and estimated the summary HR. The mean HR for the race, comparing Caucasian with Asian, was 0.90 (95% confidence interval [CI]: 0.77-1.05) for PFS and 0.93 (95% CI: 0.80-1.07) for OS (Fig. 2).

### Comparison of PFS and OS Between OECD and Non-OECD Groups

**Figure 3.**
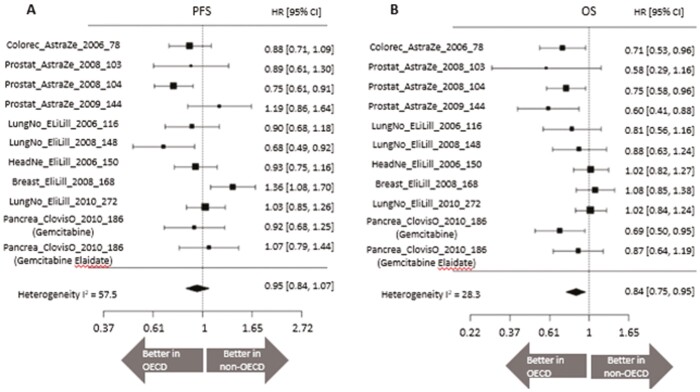
Forest plot of HRs comparing patients enrolled in the OECD and non-OECD groups for (**A**) PFS and (**B**) OS. CI, confidence interval; HR, hazard ratio; OECD, Organisation for Economic Co-operation and Development; OS, overall survival; PFS, progression-free survival..

We calculated the HR for PFS and OS between patients enrolled in the OECD group and those in the non-OECD group in each of the clinical trial data and estimated the summary HR. The mean HR, comparing patients in the OECD group with those in the non-OECD group, was 0.95 (95% CI: 0.84-1.07) for PFS and 0.84 (95% CI: 0.75-0.95) for OS (Fig. 3).

## Discussion

Our study demonstrated that patients enrolled in the OECD group showed better OS than those in the non-OECD group. Treatment drugs in each trial arm with the exception of one experimental arm were among standard medications for the targeted cancers, which drug effects are generally not considered to be impacted by intrinsic factors described in the ICH-E5 guideline (eg, genetic factors, age, gender). This was supported by the result that there was no statistically significant difference in the HRs for PFS and OS between Caucasian and Asian. Therefore, the difference in HR for OS between patients enrolled in OECD and non-OECD groups could be attributed to the influence of extrinsic factors (eg, socioeconomic factors, medical practice).

Inequalities in access to novel innovative drugs exist among countries. Global oncology trend report published from the IMS institute^17^ reported that of 49 new oncology substances, which were initially launched between 2010 and 2014 in 21 selected countries, the average number of substances available was 25.5 in the OECD group and 10.1 in the non-OECD group as of 2015. A disparity in costs of cancer treatment was also found. The “Comparator report on cancer in Europe 2019”^18^ published by the Swedish Institute for Health Economics, revealed that both the average cost for cancer care including non-drug cost (ie, medical equipment, surgery, radiation, and rehabilitation) and cancer drug sales per capita from 1995 to 2018 in the EU, were always higher in OECD group than in a non-OECD group. In addition, according to WHO Global Health Expenditure Datbase,^19^ average life expectancy at birth in OECD and non-OECD groups was 79.7 and 73.9 years old in 2010. The OECD group also spent more on healthcare per capita than the non-OECD group from 2000 to 2018 while paying a lower percentage of out-of-pocket costs. These reports indicated that the OECD group had a favorable healthcare environment and availability for innovative medicines compared with the non-OECD group.

There is a wide difference in the healthcare environment among countries, including opportunities for medical checkup, diagnosis, surgical techniques, supportive care, management of adverse events, and non-cancer deaths, which may affect both PFS and OS. Some of the factors that affected PFS are also expected to affect OS. No statistically significant difference in PFS between OECD and non-OECD groups was shown, suggesting that similar treatment effects would be expected under protocol-based management with strictly defined laboratory tests with defined frequencies. On the other hand, subsequent treatment after progression is generally performed in practice and cannot be managed by protocol. There is a high possibility that patients in OECD countries can access other treatments after disease progression and receive later treatments, as all the trials in our study were for first-line treatment. The results of our study indicated that the subsequent treatments during the PPS period carried out in practice, or in other words, the difference in accessibility to healthcare resources had an impact on the prolongation of OS.

The present study has several limitations to be mentioned. First, because it was a retrospective analysis, we did not investigate all intrinsic factors, and we only took into consideration the common data that could be collected from different types of cancers. In addition, these trials started between 2006 and 2010, and thus, genetic information, which may affect prognosis, was not included in the inclusion/exclusion criteria. Second, this study included all trials that met the criteria regardless of cancer type to collect as many trials as possible, and therefore, the type and number of subsequent treatments were different among cancers. We could not confirm directly whether each patient had post-progression treatments. Third, there is a possibility that the economic status and accessibility of drugs are not the same even among the OECD group.

The unique and strong point of the present study was that patient-level data were used in the analysis. There are several open-access data-sharing platforms, and sponsors can choose what data to publish and where. In the present study, we chose PDS, which specializes in oncology late-phase trials. Because data on products under development are difficult to be published, data on old trials would be mainly published, while publication bias due to positive or negative results of clinical trials is unlikely to occur. We divided the subjects into 2 groups based on the country where they were enrolled using individual patient data. This enabled us to focus our analysis on the socio-economic status of each country regardless of race/region. We also examined trials sponsored by different companies and explored common issues among them.

Based on the results of our study, we consider that the information about treatment provided after disease progression, the healthcare environment, and the type and number of drugs being developed and/or approved in each country is essential for the proper evaluation of OS. Including these factors in the stratification factors and capping the number of patients per region/country are options when sponsors think these factors affect the results of the trial. Clinical trial sponsors should consider carefully how to properly design oncology clinical trials including the selection of countries and data management of subsequent treatments.

## Conclusion

The present study illustrated that economic status had an impact on the outcome of OS in oncology clinical trials by re-analyzing individual patient data in past clinical trials. Prolongation of OS was observed in OECD membership countries. Clinical trial sponsors should consider carefully how to properly design oncology clinical trials including the selection of countries and data management of subsequent treatments.

## Data Availability

The data underlying this article will be shared on reasonable request to the corresponding author.

## References

[1] Imai H, Kaira K, Minato K. Clinical significance of post-progression survival in lung cancer. Thorac Cancer. 2017;8(5):379-386. 28627767 10.1111/1759-7714.12463 PMC5582459

[2] Barlesi F, Vansteenkiste J, Spigel D, et al Avelumab versus docetaxel in patients with platinum-treated advanced non-small-cell lung cancer (JAVELIN Lung 200): an open-label, randomised, phase III study. Lancet Oncol. 2018;19(11):1468-1479. 30262187 10.1016/S1470-2045(18)30673-9

[3] Herbst RS, Baas P, Kim DW, et al Pembrolizumab versus docetaxel for previously treated, PD-L1-positive, advanced non-small-cell lung cancer (KEYNOTE-010): a randomised controlled trial. Lancet. 2016;387(10027):1540-1550. 26712084 10.1016/S0140-6736(15)01281-7

[4] Rittmeyer A, Barlesi F, Waterkamp D, et al; OAK Study Group. Atezolizumab versus docetaxel in patients with previously treated non-small-cell lung cancer (OAK): a phase III, open-label, multicentre randomised controlled trial. Lancet. 2017;389(10066):255-265. 27979383 10.1016/S0140-6736(16)32517-X PMC6886121

[5] Horn L, Spigel DR, Vokes EE, et al Nivolumab versus docetaxel in previously treated patients with advanced non-small-cell lung cancer: two-year outcomes from two randomized, open-label, phase III trials (CheckMate 017 and CheckMate 057). J Clin Oncol. 2017;35(35):3924-3933. 29023213 10.1200/JCO.2017.74.3062 PMC6075826

[6] Park K, Özgüroğlu M, Vansteenkiste J, et al Impact of subsequent immune checkpoint inhibitor treatment on overall survival with avelumab vs docetaxel in platinum-treated advanced NSCLC: post hoc analyses from the phase III JAVELIN Lung 200 trial. Lung Cancer. 2021;154:92-98. 33636453 10.1016/j.lungcan.2021.01.026

[7] Sawaki A, Yamada Y, Yamaguchi K, et al Regional differences in advanced gastric cancer: exploratory analyses of the AVAGAST placebo arm. Gastric Cancer. 2018;21(3):429-438. 29058097 10.1007/s10120-017-0773-y PMC5906488

[8] Sawaki A, Ohashi Y, Omuro Y, et al Efficacy of trastuzumab in Japanese patients with HER2-positive advanced gastric or gastroesophageal junction cancer: a subgroup analysis of the Trastuzumab for Gastric Cancer (ToGA) study. Gastric Cancer. 2012;15(3):313-322. 22179434 10.1007/s10120-011-0118-1 PMC3390686

[9] Shitara K, Muro K, Shimada Y, et al Subgroup analyses of the safety and efficacy of ramucirumab in Japanese and Western patients in RAINBOW: a randomized clinical trial in second-line treatment of gastric cancer. Gastric Cancer. 2016;19(3):927-938. 26510663 10.1007/s10120-015-0559-z

[10] Vermorken JB, Stöhlmacher-Williams J, Davidenko I, et al; SPECTRUM investigators. Cisplatin and fluorouracil with or without panitumumab in patients with recurrent or metastatic squamous-cell carcinoma of the head and neck (SPECTRUM): an open-label phase III randomised trial. Lancet Oncol. 2013;14(8):697-710. 23746666 10.1016/S1470-2045(13)70181-5

[11] Song SY, Chee D, Kim E. Strategic inclusion of regions in multiregional clinical trials. Clin Trials. 2019;16(1):98-105. 30444138 10.1177/1740774518813573

[12] The International Council for Harmonization of Technical Requirements for Pharmaceuticals for Human Use (1998) Ethnic factors in the acceptability of foreign clinical data E5(R1). https://database.ich.org/sites/default/files/E5_R1__Guideline.pdf. Accessed August 1, 2021.

[13] The International Council for Harmonization of Technical Requirements for Pharmaceuticals for Human Use (2017) General principles for planning and design of multi-regional clinical trials E17. https://database.ich.org/sites/default/files/E17EWG_Step4_2017_1116.pdf. Accessed 1 August, 2021.

[14] Vrdoljak E, Bodoky G, Jassem J, et al Expenditures on oncology drugs and cancer mortality-to-incidence ratio in central and eastern Europe. Oncologist. 2019;24(1):e30-e37. 30181313 10.1634/theoncologist.2018-0093 PMC6324644

[15] Home | Share, Integrate & Analyze Cancer Research Data | Project Data Sphere. https://data.projectdatasphere.org/projectdatasphere/html/home. Accessed March 23, 2021.

[16] OECD.org - OECD. https://www.oecd.org/. Accessed March 23, 2021.

[17] Aitken M. Global Oncology Trend Report. *IMS Inst Healthc Informatics*. 2016;(June). www.theimsinstitute.org. Accessed 1 August, 2021.

[18] Hofmarcher T, Brådvik G, Jönsson B, Lindgren P, Jönsson B, Wilking N. Comparator report on cancer in Europe 2019 – disease burden, costs and access to medicines. IHE Rep. 2019:65-69. https://ihe.se/wp-content/uploads/2020/01/IHE-Report-2019_7_.pdf. Accessed 1 August, 2021.

[19] Global Health Expenditure Database. https://apps.who.int/nha/database/country_profile/Index/en. Accessed March 24, 2021.

